# High-temperature-treated soy protein isolate reduces amino acid availability and alters social novelty in mice

**DOI:** 10.1038/s41538-026-01091-0

**Published:** 2026-09-03

**Authors:** Tomoko T. Asai, Takayo Mannari-Sasagawa, Yuichi Yabe, Mami Yamada, Hitoshi Takamura, Noriko Horii-Hayashi, Satoshi Mochizuki, Mayumi Nishi, Kenji Sato

**Affiliations:** 1 Department of Food Science and Nutrition, Faculty of Human Life and Environment, Nara Women’s University, Nara, Japan; 2 Division of Applied Biosciences, Graduate School of Agriculture, Kyoto University, Kyoto, Japan; 3 Department of Anatomy and Cell Biology, Nara Medical University, Kashihara, Japan; 4 KYOUSEI Science Center for Life and Nature, Nara Women’s University, Nara, Japan; 5 Department of Food Sciences and Nutritional Health, Kyoto Prefectural University, Kyoto, Japan; 6 Faculty of Education, Oita University, Oita, Japan

**Keywords:** Biochemistry, Physiology

## Abstract

High-temperature (HT) treatments above 100 °C are important in food processing; however, their effects on the nutritional value of moist heat-treated proteins are less understood. Therefore, this study examined the impact of HT at 121 °C on soy protein isolate (SPI) in water. After 20 min of HT treatment, most amino acid levels in SPI decreased significantly, despite minimal nitrogen loss. HT also increased insoluble protein and larger soluble peptides after in vitro digestion. Mice administered HT-treated SPI showed lower serum levels of free amino acids, thereby reducing bioavailability. Long-term feeding with a 20% protein diet based on HT-treated SPI impaired social novelty recognition and decreased peripheral serotonin levels, while anxiety-like behavior, object close-range exploration, sociability, and serum tryptophan (Trp) remained unaffected. These findings indicate that HT treatment not only reduces the nutritional value of SPI but may also generate Maillard-type compounds or modified peptides that interfere with Trp metabolism and social behavior.

**Figure:**
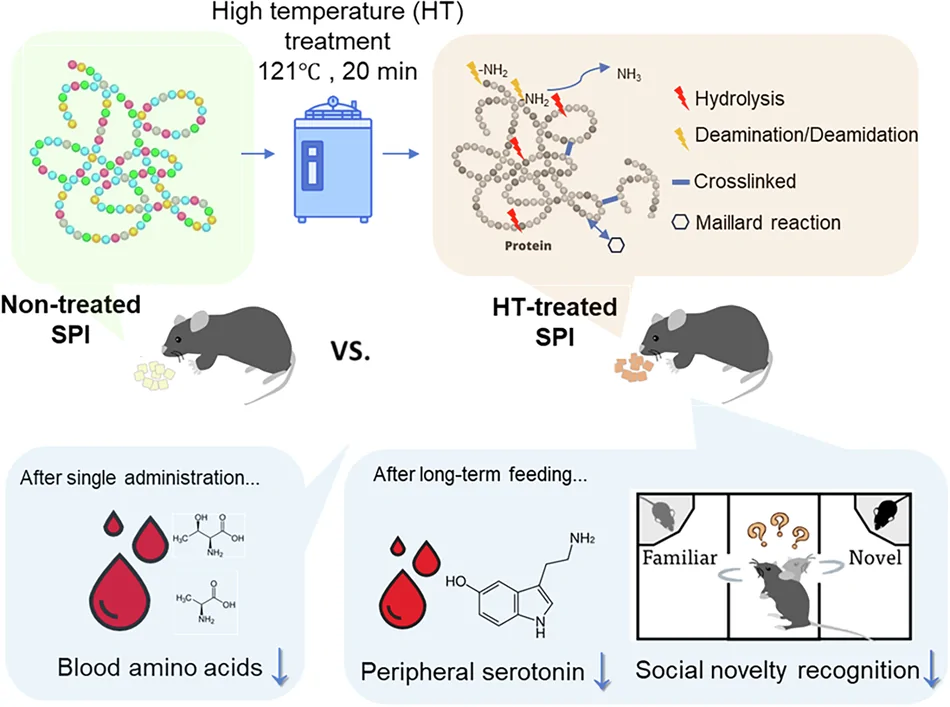

## Introduction

High-temperature (HT) treatment above 100 °C is widely used in food processing^1^. Foods packed in airtight containers, such as cans, bottles, retort pouches, and retort trays, are heated at temperatures of 121 °C or higher for an appropriate period, typically using a retort or autoclave, allowing them to be stored at room temperature. Microbial cell membranes, enzymes, and spores in foods are denatured using HT treatment, a process also known as sterilization^2^. The production of retort-packaged products is on the rise^3^, including infant foods, possibly due to no need to add synthetic preservatives. Additionally, it has been reported that protease inhibitors in foods such as soy trypsin inhibitors are inactivated by HT treatment^4^. Thus, HT treatment improves the digestibility of food protein, enhancing the nutritional value^5^. In contrast, some studies have reported that HT treatment of protein in dry form modifies amino acid residue and decreases nutritional value^6–9^. HT treatment of food proteins generates dehydroalanine residues from serine and cysteine residues, which react with lysine and cysteine residues to form lysinoalanine and lanthionine crosslinks, respectively^10^. This reaction decreases available lysine and cysteine and the digestibility of proteins. HT treatment of powdered foods, such as skim milk and soy protein isolate (SPI), in an autoclave at 121 °C has been demonstrated to decrease protein efficiency ratio and protein digestibility-corrected amino acid score in an animal model^11–13^. However, in practice, HT processing of foods is more often performed on prepared moist foods, rather than on powders^13,14^. There is only limited information on the effects of HT treatment of protein in wet form on nutritional values and their metabolic fate. In a preliminary experiment, SPI was suspended in water to achieve a 10% concentration and then heated at 121 °C for 20 min in an autoclave, simulating common food processing conditions. Rats fed a 10% protein diet in which HT-treated SPI was used as the protein source for 2 weeks exhibited significantly reduced feed efficiency compared with those fed non-treated SPI-based diet (Supplementary Tables S1 and S2), along with modest changes in serum biochemical parameters (Supplementary Table S3). In contrast, no significant change in feed efficiency was observed between the two groups when a 20% protein diet was used (data not shown). These findings prompted further investigation into the nutritional and behavioral consequences of HT-treated SPI in a mouse model. In this study, the effects of the HT treatment of SPI in the presence of water on amino acid composition, in vitro digestibility, and in vivo amino acid bioavailability were examined. Furthermore, the effects of long-term feeding of the 20% protein diet based on HT-treated SPI on the behavior of mice were examined.

## Results

### Chemical changes in HT-treated SPI

**Fig. 1:**
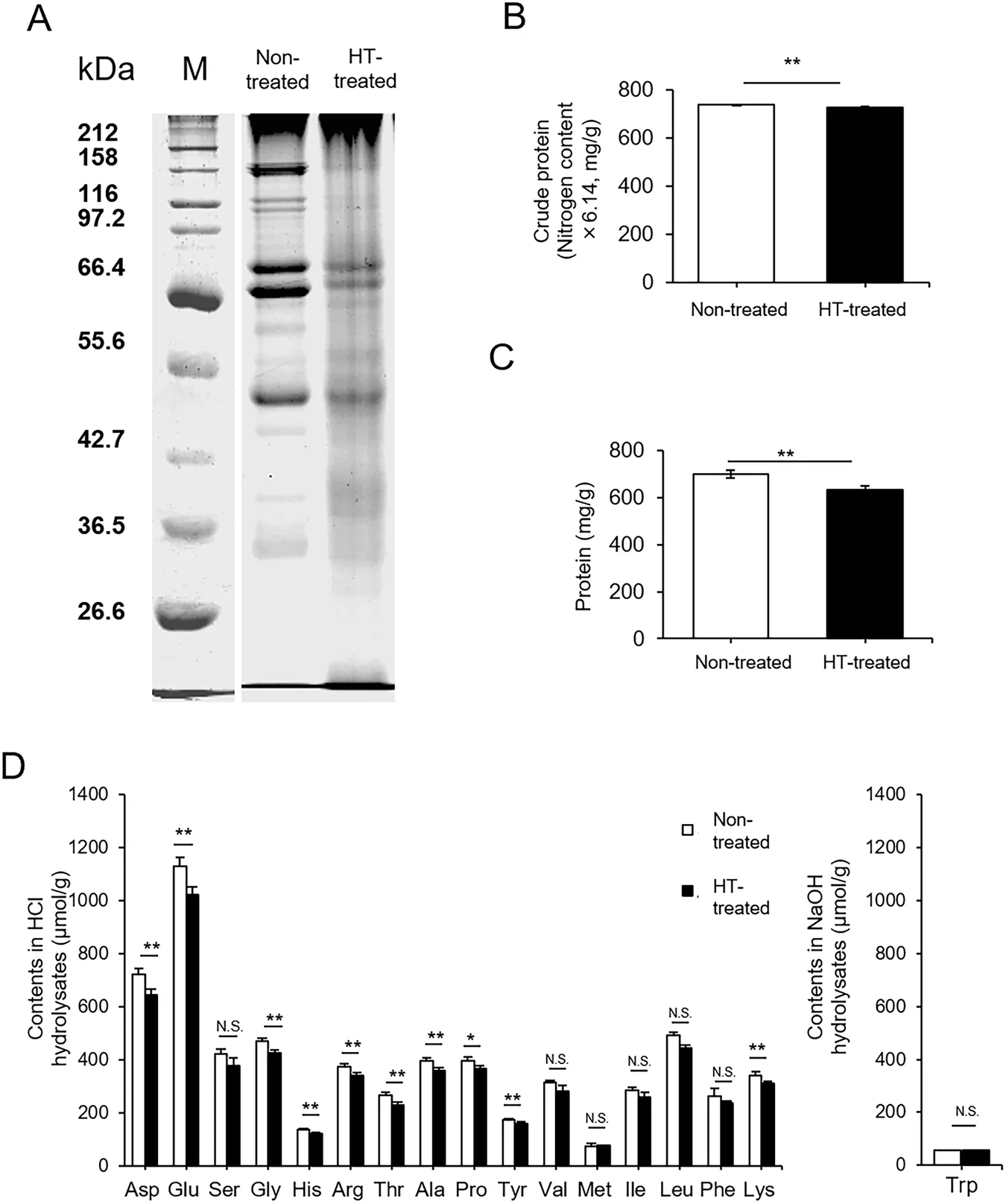
SDS-PAGE profiles, protein contents, and amino acid residues in non-treated and HT-treated SPI. **A** SDS-PAGE profiles of non-treated and HT-treated SPI. M: prestained protein molecular weight marker. **B** Crude protein content (nitrogen content × 6.14) determined using Kjeldahl analysis. **C** Protein content determined using amino acid analysis. **D** Amino acid content in HCl and NaOH hydrolysates of non-treated and HT-treated SPI. Data are presented as mean ± SD (*n* = 4–5). *P*-values were calculated using the unpaired Student’s *t*-test (*P < 0.05, **P < 0.01, ***P < 0.001).

Figure 1A shows the SDS-PAGE patterns of non-treated and HT-treated SPI. The HT-treated sample exhibited unclear protein bands and uneven staining across the gel, particularly at the bottom of the lane, suggesting that HT treatment resulted in nonspecific cleavage, aggregation, or chemical modification.

Kjeldahl analysis revealed a slight decrease in crude protein content (nitrogen content × 6.14) from 737.1 ± 0.94 mg/g for non-treated SPI to 724.6 ± 3.68 mg/g for HT-treated SPI (a decrease of approximately 1.7%, Fig. 1B). On the other hand, amino acid analysis showed a decrease in protein content from 701.90 ± 12.01 mg/g to 634.43 ± 8.29 mg/g (a decrease of approximately 9.6%, Fig. 1C). The contents of several amino acids in HCl and NaOH hydrolysates of HT-treated SPI, respectively, were significantly lower than that of non-treated SPI (Fig. 1D).

**Fig. 2:**
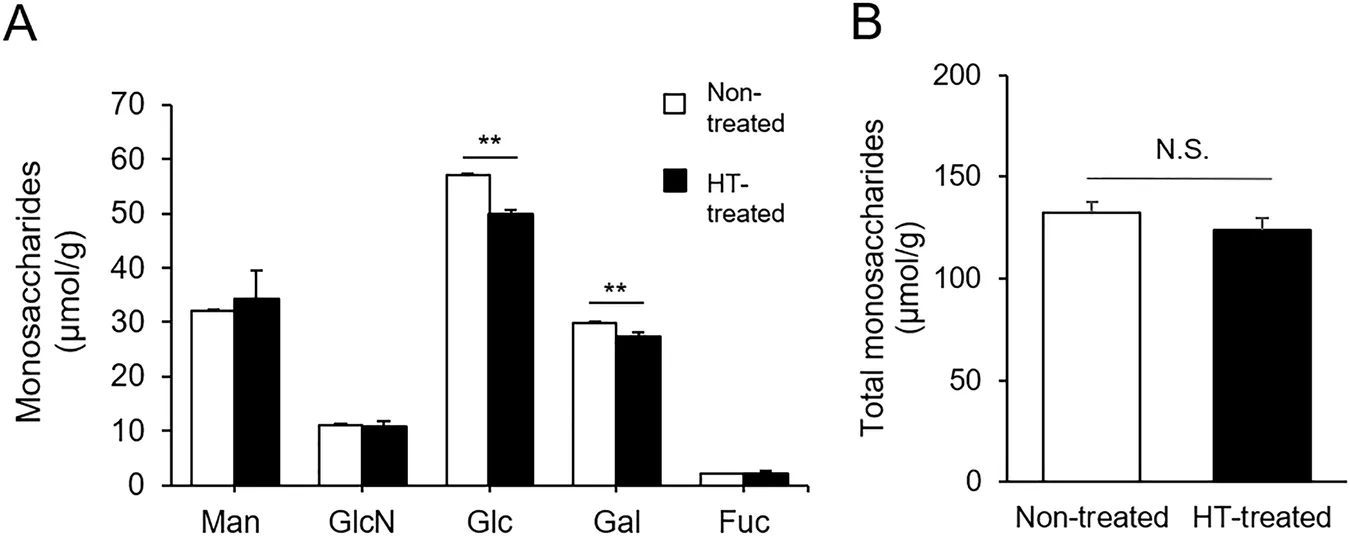
Monosaccharide composition and total sugar content in non-treated (□) and HT-treated (■) SPI. **A** Individual sugar components quantified using HPLC: Man, mannose; GlcN, glucosamine; Glc, glucose; Gal, galactose; Fuc, fucose. **B** Total monosaccharide content. Data are presented as mean ± SD (*n* = 3). *P*-values were calculated using the unpaired Student’s *t*-test (*P < 0.05, **P < 0.01).

To address the reason for the reduction of amino acid residues in SPI by HT treatment, effects of HT treatment on reducing sugars and ammonia levels were examined, as reducing sugars can react with the amino group by the Maillard reaction and ammonia can be liberated from the amino and amide groups. The contents of glucose and galactose derived from oligosaccharides such as raffinose and stachyose contaminating SPI were significantly reduced by HT treatment (Fig. 2A). No significant changes occurred in the levels of sugar components of glycoproteins, such as mannose, glucosamine, and fucose. The total reduction contents of monosaccharide in SPI by HT treatment were approximately 10 µmol/g (Fig. 2B), which was far less than the reduction of amino acids (approximately 1 mmol/g).

**Fig. 3:**
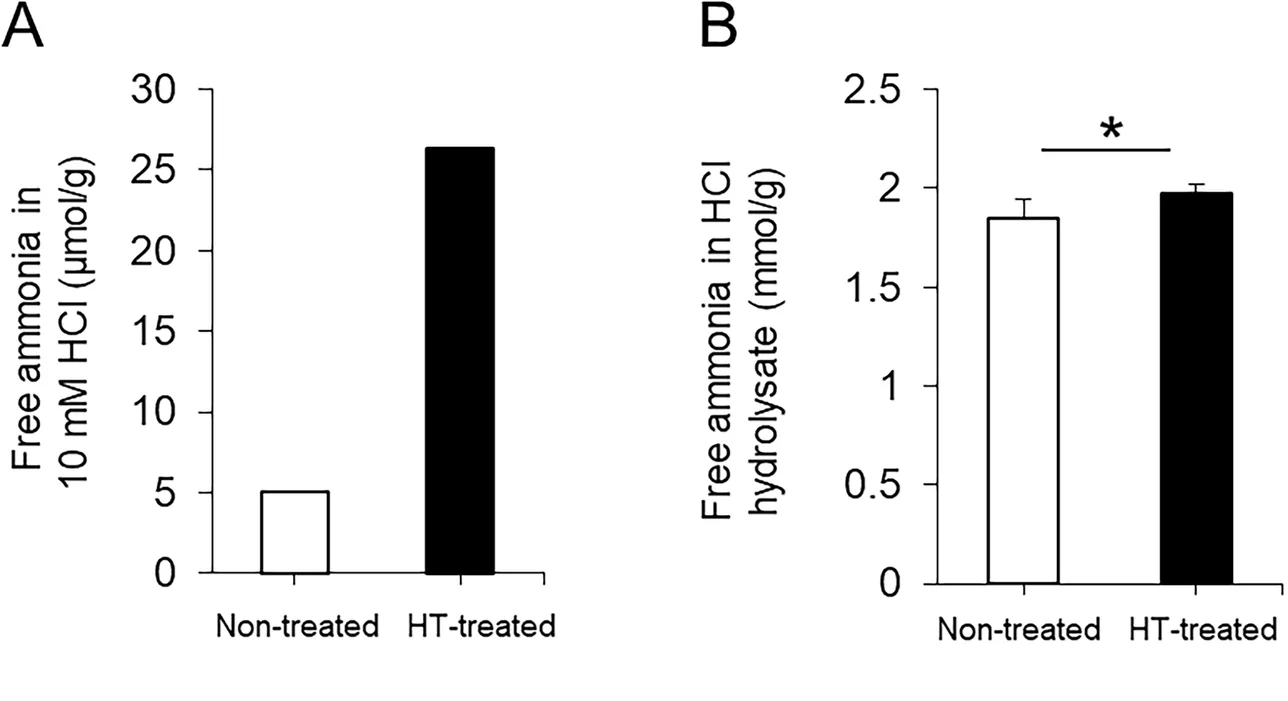
Free ammonia levels in non-treated (□) and HT-treated (■) SPI. **A** Ammonia levels before HCl hydrolysis (*n* = 1). **B** Ammonia levels after HCl hydrolysis (*n* = 3, mean ± SD). *P*-values were calculated using the unpaired Student’s *t*-test (*P < 0.05).

To investigate the contribution of deamination and deamidation reactions to the reduction of amino acid residues, the free ammonia content was quantified. Free ammonia content increased to 20 µmol/g of SPI by HT treatment (Fig. 3A), which was less than one-fiftieth of the reduction in amino acids in the hydrolysate by HT treatment (Fig. 3B). HCl hydrolysis of the non-treated SPI generated approximately 1.80 mmol of free ammonia/g of SPI. This ammonia can be derived from amide in the side chains of Asn and Gln residues in proteins. HT treatment significantly increased ammonia concentrations in the HCl hydrolysate of SPI to 1.98 mmol/g (Fig. 3B). Thus, HT treatment increased ammonia in the HCl hydrolysate by over 120 µmol/g, corresponding to approximately 12% of the loss of amino acid residues due to destruction and modification of amino acid residues by HT treatment.

### Nondigestible proteins and peptides in HT-treated SPI

**Fig. 4:**
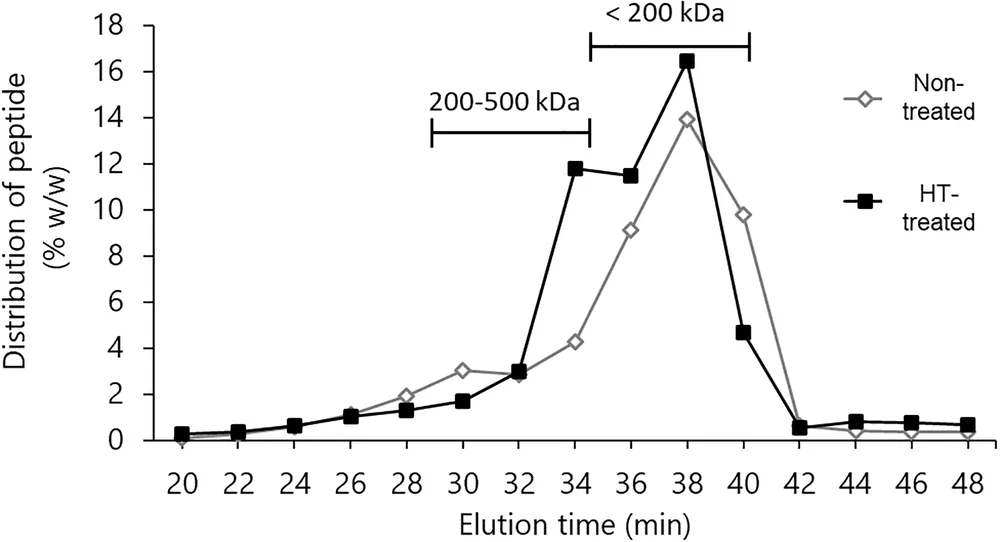
Peptide size distribution in digested SPI analyzed using size exclusion chromatography. Elution profiles of peptides from in vitro digests of non-treated (◇) and HT-treated (■) SPI. Molecular weights were estimated based on calibration standards. Before injection, samples were clarified by a 3 kDa cutoff membrane to remove remaining enzymes.

The following analysis examined how HT treatment influenced the digestibility of SPI and promoted the formation of digestion-resistant proteins and peptides. HT-treated and non-treated SPIs were digested with pepsin and pancreatin. The pepsin-pancreatin digest of non-treated SPI was completely soluble, whereas the digest of HT-treated SPI contained 53.0 ± 9.9 mg of insoluble material/g of SPI (*n* = 3). Amino acid analysis revealed that insoluble material contained protein (Supplementary Fig. S1). This indicates that HT treatment generates insoluble proteins that are resistant to endoproteinases. To assess the resistance to exopeptidases, the soluble fractions of the pepsin-pancreatin digest were further digested with exopeptidases using carboxypeptidase A and leucine aminopeptidase. The peptides in the endoproteinases-exopeptidases digests of HT-treated and non-treated SPI were analyzed using size exclusion chromatography (SEC). Elution of the peptide was monitored using amino acid analysis of the HCl hydrolysate of the SEC fraction (Fig. 4). HT treatment of SPI increased the ratio of peptides with molecular weights ranging from 200 to 500 Da in the digest, while decreasing peptides with molecular weights less than 200 Da. These results indicate that HT treatment modifies the amino acid residues of proteins in SPI, resulting in the formation of digestion-resistant peptides that remained even after exopeptidase treatment.

### Mice serum amino acids levels after single administration of SPIs

**Fig. 5:**
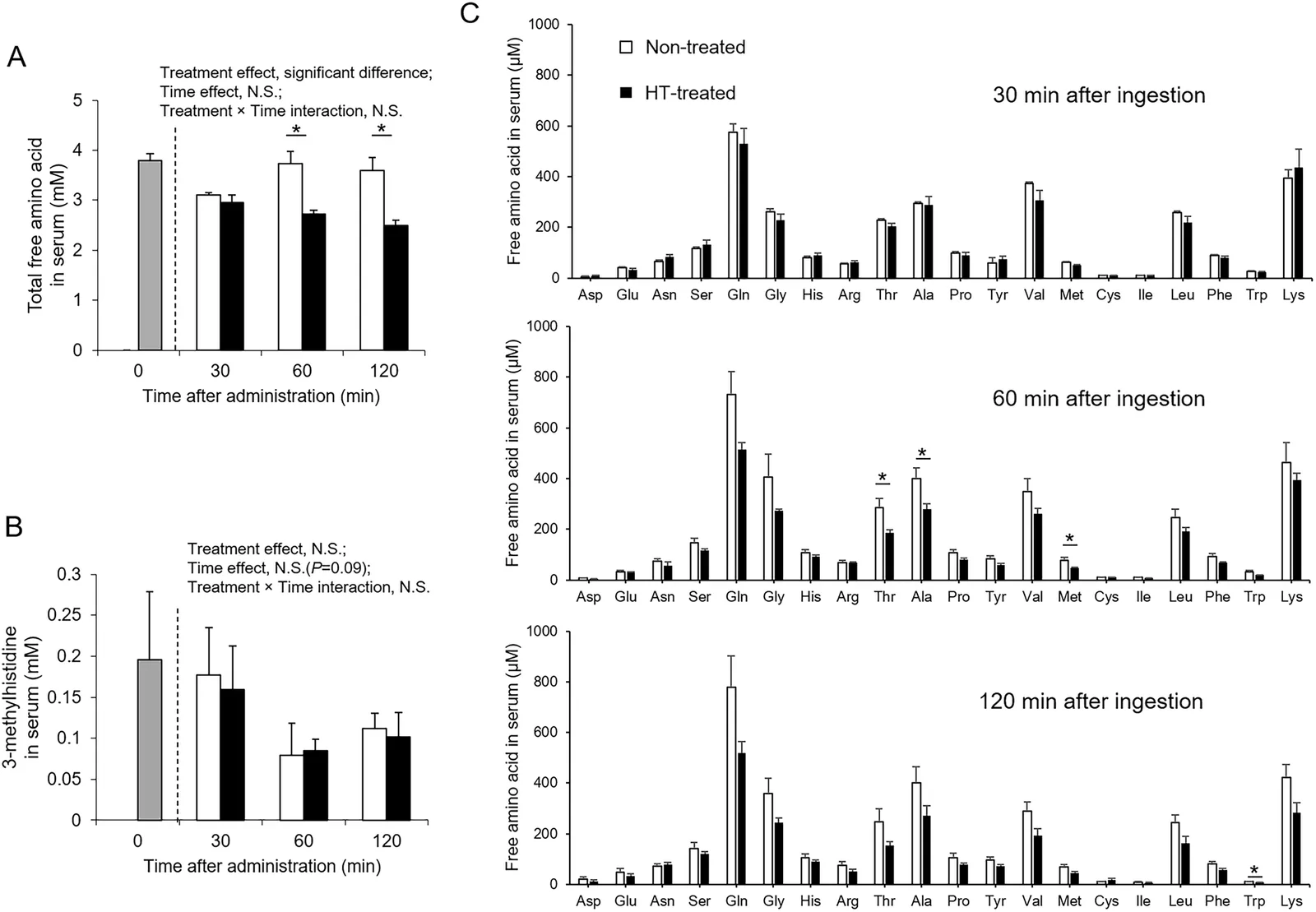
Serum free amino acid and 3-methylhistidine levels after single oral administration of SPI. **A** Serum concentrations of total free amino acids. **B** Serum concentrations of 3-methylhistidine. **C** Serum concentrations of individual free amino acids. Baseline values in (**A**, **B**) were obtained from fasted mice before SPI administration and are shown as a common pre-administration control. Because serum collection required terminal sampling, a single baseline group was used to minimize animal use. Serum samples after administration were collected at 30, 60, and 120 min after oral gavage of non-treated or HT-treated SPI. Data are presented as mean ± SEM (*n* = 4 per group). ■, baseline; □, non-treated SPI; ■, HT-treated SPI. Temporal changes from 30 to 120 min in (**A**, **B**) were analyzed using two-way ANOVA. Group differences between non-treated and HT-treated SPI at each post-administration time point in (**A**, **B**), and for individual amino acids in (**C**), were analyzed using the unpaired Student’s *t*-test. *P < 0.05, **P < 0.01, ***P < 0.001.

The effects of HT-treated SPI administration on temporal changes in serum free amino acid concentrations were subsequently investigated. Figure 5A shows the contents of total free amino acid in mouse serum at baseline after an 18 h overnight fasting, 30, 60, and 120 min after administration of non-treated SPI or HT-treated SPI. Neither administration of non‑treated nor HT‑treated SPI increased serum total amino acid levels compared with baseline. Therefore, 3‑methylhistidine, an indicator of muscle protein breakdown^15^, was quantified as shown in Fig. 5B. Two-way ANOVA indicated a tendency for serum 3-methylhistidine levels to decrease after administration of both samples (*P* = 0.09), suggesting that SPI administration may suppress muscle protein breakdown. No significant difference in serum 3-methylhistidine levels was observed between the two groups. In contrast, serum total free amino acid concentrations were significantly lower in the HT-treated SPI group than in the non-treated SPI group at 120 min. As shown in Fig. 5C, serum levels of Thr, Ala, and Met were significantly lower 60 min after administration of HT-treated SPI than those of the non-treated SPI. Trp levels were significantly lower at 120 min after administration of HT-treated SPI. In contrast, no significant differences were observed in serum amino acid concentrations between mice administered with HT-treated SPI and those with non-treated SPI at 30 min after administration.

### Effects of HT-treated SPI feeding on behavior

Behavioral outcomes were evaluated after a 6-week feeding period using the experimental diets described in Table 1, in which casein in the standard AIN-93G formulation was replaced with either non-treated SPI or HT-treated SPI as the sole protein source. No significant differences in body weight were observed between mice fed non-treated SPI and those fed HT-treated SPI at any time point during the intervention period (Supplementary Fig. S2).

**Table 1:** Composition of experimental diets

Components (g)	Non-treated SPI	HT-treated SPI
Cornstarch	39.7	39.7
Non-treated SPI	20.0	-
HT-treated SPI	-	20.0
Alfa-corn starch	13.2	13.2
Sucrose	10.0	10.0
Soybean oil	7.0	7.0
Cellulose	5.0	5.0
Mineral mixture	3.5	3.5
Vitamin mixture	1.0	1.0
Choline tartrate	0.25	0.25
L-cystine	0.3	0.3
*t-*Butylhydroquinone	0.0014	0.0014
Total	100	100

**Fig. 6:**
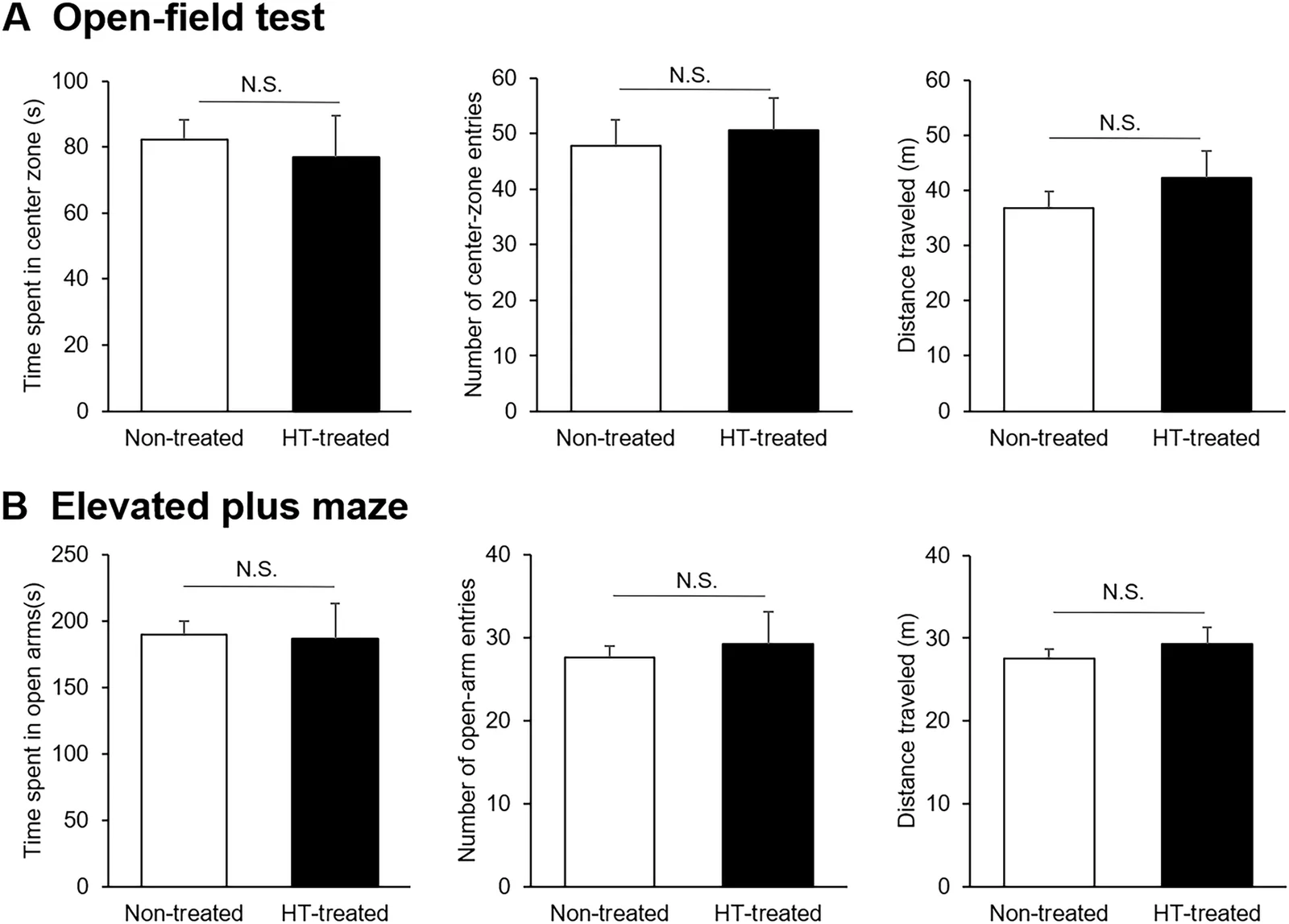
Anxiety-like behavior in mice after long-term feeding of SPI. Behavioral assessment using the (**A**) open-field test and (**B**) elevated plus maze test. Data are presented as mean ± SEM (*n* = 12 per group). *P*-values were calculated using the Mann–Whitney U test. N.S. not significant.

**Fig. 7:**
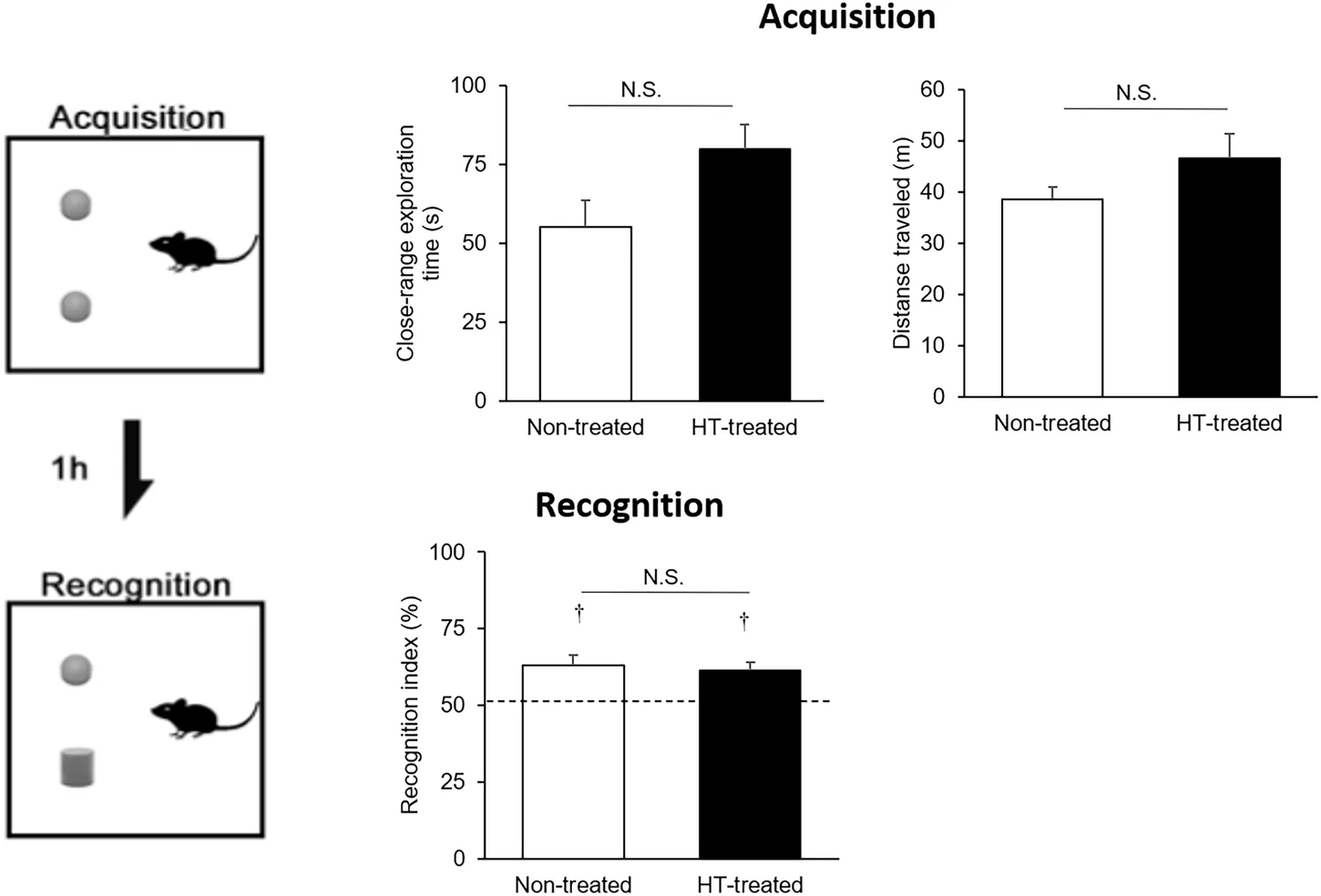
Object recognition memory in mice after long-term SPI feeding. Object recognition test results in mice fed non-treated SPI (□) or HT-treated SPI (■) (*n* = 12 per group). (Upper) Total close-range exploration time and distance traveled during acquisition. (Lower) Object recognition index. Chance level (50%) was evaluated using a one-sample *t*-test, and significant deviations from chance are indicated by †. Group differences in acquisition measures were analyzed using the Mann–Whitney U test, whereas the recognition index was compared between groups using an unpaired *t*-test. Data are presented as mean ± SEM. N.S. not significant.

Anxiety-like behavior was assessed using the open-field test and the elevated plus maze test. In both tests, no significant differences were observed between the two groups in terms of time spent in the center zone or on the open arms, the number of entries, or total locomotor activity (Fig. 6). In the object recognition test, no significant differences were found between the groups in close-range exploration time on the objects or in locomotor activity during the acquisition session (Fig. 7). In the recognition session, mice from both groups spent significantly longer close-range exploration time on the novel object than on the familiar one, and their recognition indices were significantly higher than the chance level (50%). The recognition index did not differ significantly between the two groups.

**Fig. 8:**
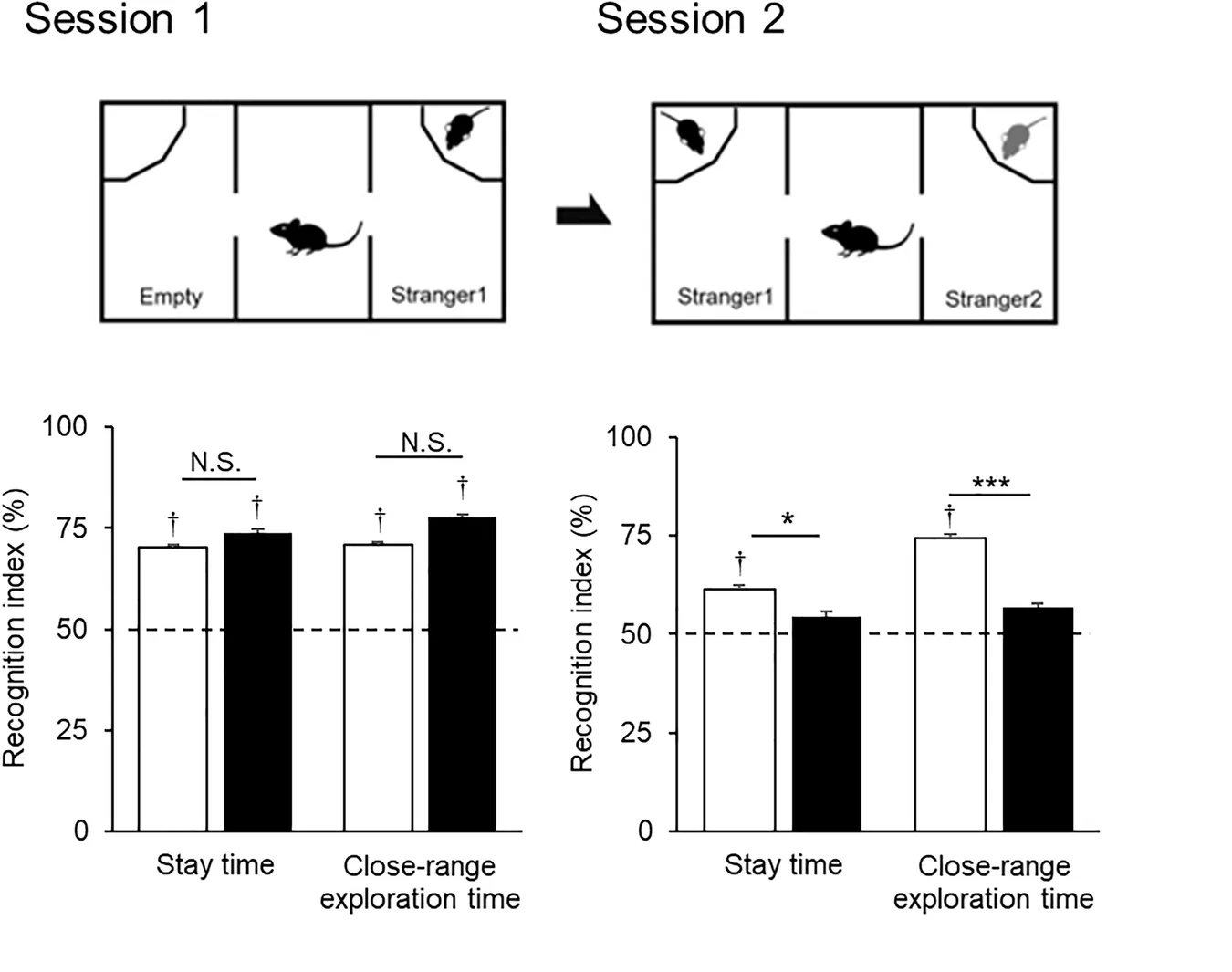
Social behavior in mice after long-term SPI feeding. Three-chamber social test. (Session 1) Mice explored an empty cage or a cage with Stranger 1. (Session 2) Stranger 1 was moved to the opposite chamber and Stranger 2 was introduced. The sociability index and social novelty recognition index were calculated based on chamber stay time and close-range exploration time. Group differences in these indices were analyzed using the Mann–Whitney U test. Chance level (50%) was evaluated using a one-sample t-test, and significant deviations from chance are indicated by †. Data are presented as mean ± SEM (*n* = 12 per group). □, non-treated SPI; ■, HT-treated SPI. *P < 0.05, **P < 0.01, ***P < 0.001, ****P < 0.0001.

To evaluate social behavior, the three-chamber social test was performed (Fig. 8). In session 1 (the sociability phase), the sociability recognition indices (the ratio of stay time or close-range exploration time in the chamber containing Stranger 1 to that in the empty chamber) were significantly higher than the chance level (50%) in both groups. No significant differences were observed between the two groups in any of the parameters during session 1. In session 2 (the social novelty phase), only mice fed non-treated SPI showed significantly higher sociability recognition indices (ratio of stay time or close-range exploration time in the chamber containing Stranger 2 to that in the chamber containing Stranger 1). Significant differences in the sociability recognition index were observed between the two groups during session 2.

### Effects of HT-SPI feeding on serotonin and Trp levels

Serum and whole brain Trp and serotonin levels were assessed after the same 6-week feeding period with experimental diets containing either non-treated SPI or HT-treated SPI as the protein source.

**Fig. 9:**
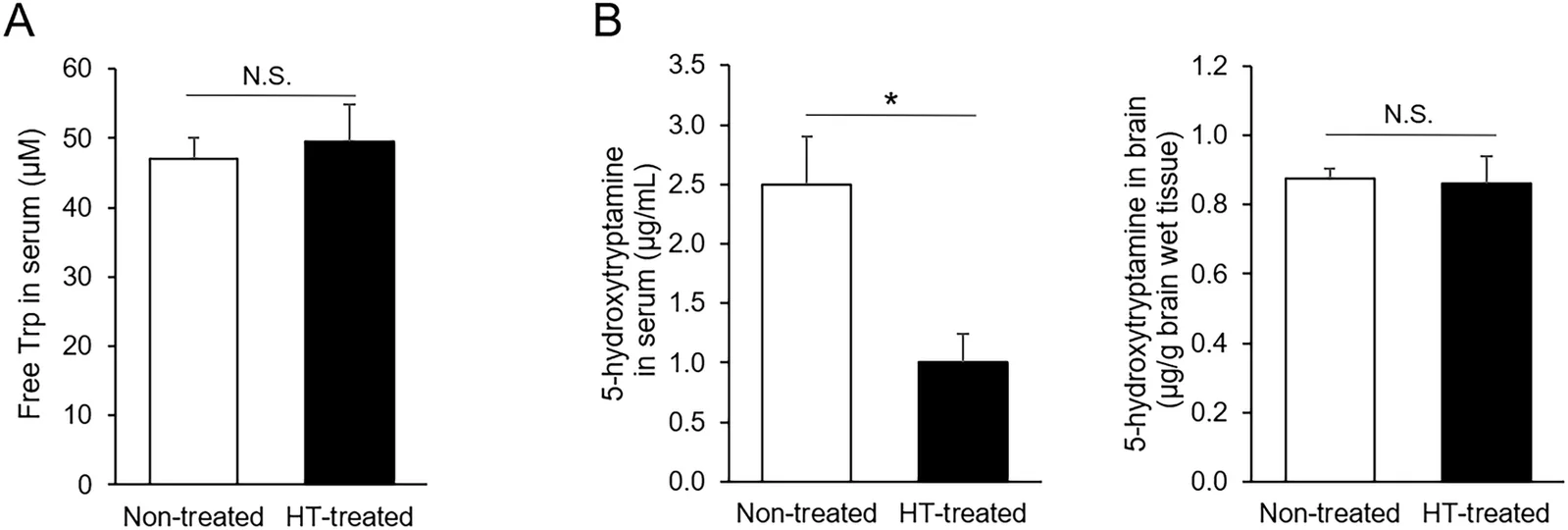
Serum Trp and serotonin levels and brain serotonin levels after long-term SPI feeding. **A** Serum free Trp levels in mice fed non-treated SPI (□) or HT-treated SPI (■) (n = 7 per group). **B** Serum serotonin (5-hydroxytryptamine) levels (n = 7 per group) and whole-brain serotonin levels (n = 4 per group) in mice fed non-treated SPI (□) or HT-treated SPI (■).

After 6 weeks of feeding experimental diets, serum Trp levels and whole brain serotonin levels showed no significant difference between mice fed HT-treated SPI and those fed non-treated SPI. In contrast, serum serotonin levels were significantly lower in HT-treated SPI-fed mice than in non-treated SPI-fed mice (Fig. 9).

## Discussion

High-temperature (HT) treatment above 120 °C is widely used in food processing, particularly for sterilizing moist foods such as canned, bottled, and retort-pouched products^1–3^. The chemical and nutritional consequences of such moist HT treatment remain less well characterized despite extensive reports on dry heating effects^7,8^. In this study, we used a 10% aqueous suspension of SPI heated at 121 °C for 20 min to model moist heat sterilization and to evaluate its effects on amino-acid composition, digestibility, and biological consequences.

SPI contains approximately 90% protein and 4–6% carbohydrates and minerals per dry matter. Under the present conditions, HT treatment resulted in a ~10% reduction in amino acid residues, to a level similar to that observed with dry heating, while nitrogen loss was limited to only 1.3% (Fig. 1B, C). This discrepancy suggests that most amino acid residues were chemically modified rather than volatilized. Dry heating has been reported to liberate ammonia from proteins^12^. To minimize ammonia loss before analysis, SPI samples were sealed during heating and immediately frozen. HCl was added to the frozen samples to stabilize volatile compounds. Nonetheless, a small amount of nitrogen was lost, suggesting the formation and loss of volatile nitrogen compounds such as pyrazines, which are poorly ionized even in acid^16^. However, the sum of nitrogen loss and ammonia production by HT treatment accounted for only approximately 15% of the loss of the total amino acid residue (Figs. 1 and 3), suggesting that most lost residues may have been converted into non-volatile nitrogen compounds. Hydrolysis of proteins with HCl releases ammonia from the acid amides of Asn and Gln residues. HT treatment liberated an additional 0.2 mmol/g of ammonia during HCl hydrolysis of SPI, equivalent to approximately 20% of the loss of amino acid residues. This suggests that HT treatment induces the formation of amides or imines, such as Schiff bases, which can release ammonia through acid hydrolysis. Since reducing sugars in SPI were decreased by HT treatment (Fig. 2), carbonyl compounds generated by the Maillard reaction may have reacted with amino groups to produce imines.

The present study revealed that most of the amino acid residues were significantly reduced by HT treatment of SPI. Heat and alkali treatments of proteins are known to induce the formation of lysinoalanine or lanthionine from specific residues such as lysine, cysteine, and serine, which decreases the availability of essential amino acids. SDS-PAGE analysis of HT-treated SPI showed reduced staining intensity and a smeared lane (Fig. 1A), a pattern consistent with extensive chemical modification, aggregation, or partial peptide bond cleavage, rather than a single specific reaction. Taken together with the broad loss across many amino acid residues (Fig. 1D), these observations are most consistent with partial hydrolysis of protein that liberates amino groups; some liberated amino groups likely underwent secondary reactions to form amides, imine type adducts (e.g., Schiff bases), or crosslinks such as lysinoalanine and lanthionine. Such nonspecific hydrolysis, combined with secondary modifications, can explain reductions in residues beyond those with reactive side-chain amino groups (for example, Lys). Furthermore, we hypothesize that other chemical changes, such as oxidation and decarboxylation^9,17^ of amino acid residues, may occur during HT treatment and contribute to the loss of amino acid residues and the production of digestion-resistant products; however, no such reactions have been reported during moist heating of protein.

HT treatment generated insoluble protein in SPI after in vitro digestion with pepsin and pancreatin. Additionally, HT treatment increased the proportion of exopeptidase-indigestible peptides in the soluble fraction of the pepsin-pancreatin digest (Fig. 4). These observations indicate that HT treatment promotes the formation of both insoluble and soluble digestion-resistant products. Amino-acid analysis showed that these resistant products still contain normal amino acids, since HCl hydrolysis released expected amino-acid profiles from both insoluble and soluble fractions (Supplementary Fig. S1 and Fig. 4). Consequently, the amount of non-bioavailable amino acids in HT-treated SPI likely exceeds the portion directly destroyed by HT treatment (≈10%). Consistent with impaired immediate bioavailability, total serum free amino-acid levels 60 and 120 min after oral administration of HT-treated SPI were ~25% lower than after non-treated SPI (Fig. 5A). In preliminary feeding trials, rats given a 10% protein-restricted diet containing HT-treated SPI for 2 weeks showed reduced feed efficiency compared with rats fed the non-treated SPI diet (Supplementary Table S2), consistent with loss of available amino acids after HT treatment. Similar reductions in protein digestibility have been reported for other heating conditions, including studies on dry-heated SPI in broilers^8,18,19^ and human diets rich in Maillard reaction products^20^. The acute oral administration study was designed to evaluate early postprandial serum amino acid responses after a defined dose of SPI, and does not reproduce the long-term dietary exposure used in the behavioral study. Although mouse age may influence digestion and absorption, systematic acute administration experiments at multiple ages were beyond the scope of this study. Therefore, these data should be interpreted as supporting evidence for reduced digestibility and amino acid availability of HT-treated SPI.

It has been reported that N‑terminal glutamine residues in peptides can undergo intramolecular cyclization between the amino group and the side‑chain amide to form a pyrrolidone structure (pyroglutamyl residue; pyroGlu) during heat processing^21^. Oligopeptides containing pyroGlu residues are resistant to exopeptidase digestion^22^. In addition to pyroglutamyl peptides, peptides bearing other HT-induced modifications—such as deamidation, deamination, decarboxylation, or crosslink formation—may also become resistant to proteolytic digestion. HT treatment has been reported to alter protein allergenicity by changing epitope structures, with possible increases or decreases in allergenic potential^7,23^. Although allergenicity was not examined in the present study, the generation of chemically modified, digestion-resistant peptides raises the possibility of altered allergenicity and warrants further immunological evaluation.

In a 7‑week feeding study using a 20% protein diet, mice fed HT‑treated SPI did not show reduced body‑weight gain compared with those fed non‑treated SPI (Supplementary Fig. S2), likely because the total dietary protein level (20%) remained sufficient to support normal growth. However, mice given HT‑treated SPI exhibited a selective impairment in social novelty recognition (Fig. 8), whereas object recognition and anxiety‑like behaviors were unaffected (Figs. 6 and 7). In the present study, whole‑brain serotonin levels did not differ significantly between groups. Nevertheless, region‑specific alterations in serotonin—such as in the prefrontal cortex, amygdala, or hippocampus—may underlie the observed change in social novelty recognition but would not be detectable in whole‑brain homogenates^24^. Alternatively, peripheral rather than central serotonin may contribute to the selective behavioral phenotype. Indeed, it has been reported that peripheral‑serotonin‑deficient rats generated by knockout of peripheral Trp hydroxylase 1 (TPH1) exhibit anxiety‑like behavioral changes despite preserved central serotonin levels^25^. This supports a functional link between reduced peripheral serotonin and behavioral modulation. The previous study^25^ used animals with near‑complete depletion of peripheral serotonin, whereas the reduction observed in the present study was partial (approximately 50% of control). Such a partial decrease may selectively affect behaviors related to social novelty without influencing anxiety‑like behavior. Differences in the availability of serotonin synthesis intermediates may also contribute, because TPH1 catalyzes the first step of serotonin synthesis from Trp^26^.

Peripheral serotonin is synthesized by enterochromaffin cells in the gut. In the present study, fasting serum Trp levels did not differ between groups (Fig. 9), suggesting that reduced Trp availability is unlikely to explain the decrease in peripheral serotonin. Instead, impaired serotonin synthesis in enterochromaffin cells represents a plausible mechanism. Yano et al. reported that serotonin synthesis by enterochromaffin cells can be modulated by gut microbial metabolites^27^. Accordingly, modified amino acids or digestion‑resistant peptides generated by HT treatment of SPI or their microbial metabolites—may influence enterochromaffin cells serotonin synthesis and related amino‑acid metabolic pathways. Taken together, these findings support the interpretation that HT‑induced chemical modification of SPI reduces amino‑acid bioavailability and generates modified peptides that may alter peripheral serotonin regulation, thereby contributing to the selective impairment in social novelty recognition.

In conclusion, this study demonstrated that moist HT treatment (121 °C, 20 min) of SPI reduced the recoverable total amino-acid content after HCl hydrolysis by approximately 10%. HT treatment also significantly increased free ammonia and ammonia released by HCl hydrolysis, consistent with deamination, deamidation, and imine formation via Maillard reaction. These modifications promoted the formation of poorly digestible peptides and insoluble protein products, further reducing the nutritional value of SPI. Moreover, long-term administration of HT-treated SPI to mice decreased peripheral serotonin levels and was associated with impaired social cognitive behavior. Because moist HT treatment is widely used in the food industry for canned, bottled, and retort-pouched products—including infant formula—further research is needed to (i) elucidate the chemical structures of modified amino acids and peptides formed by HT treatment, (ii) determine their effects on digestion, microbiota, and enteroendocrine function, and (iii) assess the impact of consuming HT-treated foods on human nutrition, development, and behavior.

## Methods

### Preparation of HT-treated SPI

SPI (Fujipro new-E, contains approximately 90% protein and 4–6% carbohydrates and minerals per dry matter) was a kind gift from Fuji Oil (Osaka, Japan). SPI was mixed with distilled water to a final concentration of 10% (w/w). The paste (10 mL, pH 7.12) was sealed in a 40 mL vial (CV-400, Pierce, Thermo Fisher Scientific, Waltham, MA, USA) and heated at 121 °C for 20 min in an autoclave (SS-320; Tomy Seiko, Japan). The products were then freeze-dried and powdered. The powder was used as an HT-treated SPI.

### Chemicals

Mineral mixture (AIN-93-MX) and vitamin mixture (AIN-93-VX) were purchased from MP Biomedicals (Solon, OH, USA). Amino acids standard mixture (Type H), 3-Methylhistidine, acetonitrile (high-performance liquid chromatography (HPLC)-grade), triethylamine, phenyl isothiocyanate, ammonium acetate, trifluoroacetic acid (TFA), choline tartrate, and *t-*butylhydroquinone were purchased from Fuji Film Wako Pure Chemical (Osaka, Japan). Soybean oil, cellulose, alfa-corn starch, sucrose, L-cystine, pancreatin, and thymol were purchased from Nacalai Tesque (Kyoto, Japan). Porcine pepsin, leucine aminopeptidase (cytosolic; porcine kidney), and carboxypeptidase A (bovine pancreas) were purchased from Merck (Darmstadt, Germany). 1-Phenyl-3-methyl-5-pyrazolone (PMP) was obtained from Dojindo (Kumamoto, Japan). 6-Aminoquinolyl-*N*-hydroxysuccinimidyl carbamate (AQC) was purchased from Toronto Research Chemicals (Toronto, ON, Canada). All other reagents were of analytical grade or better.

### In vitro protease digestion

To detect indigestible peptides generated by HT treatment, non-treated and HT-treated SPI were subsequently digested by pepsin, pancreatin, and exopeptidases^28^. One gram of SPI and HT-treated SPI were suspended in 150 mL of 0.1 M HCl. Ten milligrams of pepsin were added and incubated at 37 °C for 3 h. The reaction mixture was mixed with 10 mL of 1 M Tris-HCl buffer, pH 8.0, and the pH was adjusted to 8.0 by adding drops of 1 M NaOH. Forty milligrams of pancreatin were added and incubated at 37 °C for 24 h. The pepsin-pancreatin digest was clarified through centrifugation at 5000 × *g* for 15 min. The residue was washed twice with water and then freeze-dried. The supernatant was diluted to 500 mL with water and used as a pepsin-pancreatin soluble sample. Leucine aminopeptidase (5 µL) and carboxypeptidase A (5 µL) were added to aliquots of the supernatant (500 μL, adjusted at pH 7.5) and incubated at 37 °C for 24 h. Enzymes in the digest including trypsin (23–24 kDa) were removed by passing the sample through an Amicon Ultra 0.5 mL 3 kDa molecular weight cutoff filter (Merck) using centrifugation at 12,000 × *g* for 20 min.

### Size exclusion chromatography

Aliquots of peptidase digest were clarified by passing a Cosmonice filter (0.45 μm, Nacalai Tesque). Two hundred microliters of the filtrates were subjected to size exclusion chromatography (SEC) using a Superdex Peptide 10/300 GL (GE Healthcare, Chicago, IL, USA). Elution was performed using 0.1% TFA containing 30% acetonitrile at a flow rate of 0.5 mL/min. Fractions were collected every 1 min. Fractionation was repeated three times, yielding 1.5 mL of each fraction.

### Amino acid analysis

Non-treated SPI, HT-treated SPI, and insoluble residue samples obtained after pepsin–pancreatin digestion (10.0 mg dry weight per sample) were subjected to acid hydrolysis. Samples were placed in 40-mL Pierce vials (CV-400; Thermo Fisher Scientific) fitted with Mininert^®^ valves. Then, 1.0 mL of 6 M HCl was added, the vial atmosphere was evacuated using a vacuum pump, the valve was closed, and the vials were hydrolyzed at 150 °C for 1 h. After cooling, hydrolysates of bulk samples were diluted to 10.0 mL with Milli-Q water, filtered (0.45 µm), and aliquots (20 µL) were processed for amino-acid derivatization and HPLC analysis according to Bidlingmeyer et al.^29^ with slight modifications^30^. Due to the instability of cysteine and cystine during acid hydrolysis and the difficulty of quantitative performic acid oxidation for insoluble samples, cysteine and cystine contents were not determined. The total nitrogen content of proteins was measured using the Kjeldahl method.

Aliquots of SEC fractions were processed using the vapor phase hydrolysis method. The samples were first dried in glass tubes (50 × 4 mm i.d.), then transferred into 40-mL Pierce vials containing 1.0 mL of 6 M HCl. The vial atmosphere was evacuated using a vacuum pump, the valve was closed, and the samples in the inner tubes were hydrolyzed by exposure to HCl vapor at 150 °C for 1 h. The resulting hydrolysates were then reconstituted and analyzed as described above.

Trp in protein was measured after alkaline hydrolysis; SPI and HT-treated SPI were hydrolyzed under alkaline conditions, and Trp content was quantified using reversed-phase HPLC at Japan Food Research Laboratories (Tokyo, Japan). Free amino acids in SEC fractions and mouse serum were determined after protein removal: aliquots were mixed with three volumes of ethanol, centrifuged at 10,000 × *g* for 5 min. The supernatant was then separated and subjected to amino acid analysis as described above without hydrolysis.

To estimate breakdown of muscle proteins, 3-methylhistidine was determined. Aliquots of 75% ethanol soluble fractions (20 µL) were derivatized with AQC by the method as described previously^31^. The derivatives were resolved by liquid chromatography-tandem mass spectrometer (LC-MS/MS) using an LCMS-8040 (Shimadzu) and Inertsil ODS-3 column (2.1 mm i.d. × 250 mm; GL Science, Tokyo, Japan). Binary linear gradient was performed using 0.1% formic acid (solvent A) and 0.1% formic acid containing 80% acetonitrile (solvent B) at a flow rate of 0.2 mL/min. The gradient program was as follows: 0−20 min, 0−30% B; 20−25 min, 30−100% B; 25−30 min, 100% B; 30−40 min, 0% B. The column was maintained at 40 °C. Detection was performed using a multiple reaction monitoring mode optimized for the protonated divalent ions of standard AQC-3-methylhistidine.

### Reducing sugar analysis

Sugars in the sample were hydrolyzed with 2.5 M TFA. The sample (10 mg) was placed into the 40-mL vial with 2 mL of 2.5 M TFA and hydrolyzed under vacuum at 100 °C for 6 h. The hydrolysate was made up to 5 mL with water and used as a sample. Standard solutions of monosaccharide (100 mM in water) were freshly prepared and further diluted with 75% ethanol to final concentrations of 2.5 mM and derivatized with PMP using the method of Honda et al.^32^ PMP was dissolved in methanol (0.5 M) and mixed with the same volume of 0.3 M NaOH. Samples and standard solutions (10 μL) were dried in a tube (1.5 mL) and then mixed with 60 μL of a PMP−alkaline mixture and heated at 70 °C for 30 min. The reaction was terminated by adding 200 μL of 0.1 M HCl and 200 µL of 100 mM ammonium acetate buffer, pH 6.0. To remove the non-reacted PMP, liquid−liquid partitioning was performed. Chloroform (200 μL) was added, the mixture was vortexed vigorously, and then centrifuged at 10,000 × *g* for 5 min. The aqueous layer was collected and filtered using a Cosmonice Filter before HPLC analysis. The derivatives in 10 μL of filtrate were separated using a Superspher 100 RP-18 (e) (4 µm, Merck) at 45 °C using a binary gradient. Solvent A consisted of 150 mM ammonium acetate buffer, pH 6.0, containing 5% (v/v) acetonitrile; solvent B was 60% (v/v) acetonitrile. The gradient profile was as follows: 0–5 min, B 25%; 5–20 min, B 25–30%; 20–30 min, B 100%; 30–43 min, B 25%. The flow rate was 0.6 mL/min. Elution was monitored by measuring absorbance at 254 nm.

### Sodium dodecyl sulfate–polyacrylamide gel electrophoresis (SDS-PAGE) analysis

SPI and HT-treated SPI samples (10 mg dry weight each) were mixed with 1 mL of Laemmli sample buffer containing β-mercaptoethanol and heated at 95 °C for 5 min. Equal volumes of each sample solution corresponding to equal dry weight amounts of SPI were loaded onto each lane for SDS-PAGE analysis. Proteins were separated on 12% polyacrylamide gels under reducing conditions using standard SDS-PAGE protocols^33^. Electrophoresis was performed at 120 V for 60 min, followed by staining with Coomassie Brilliant Blue R-250. The gel was scanned using a Masterscan (Scanalytic, Billerica, MA, USA) using reflected‑light scanning mode. A pre-stained protein molecular weight marker (TEFCO, Tokyo, Japan) was run on the same gel for molecular weight estimation. Irrelevant lanes were removed from the final figure for clarity.

### Ammonia quantification

SPI (100 mg) was suspended in 1 mL of distilled water and autoclaved in a sealed polypropylene microtube at 121 °C for 20 min. To prevent ammonia evaporation, the sealed sample was frozen, and then 100 μL of 1 M HCl was added to the frozen sample. After melting the frozen sample, ammonia in the sample was converted to ammonium chloride. Aliquots (100 μL) of this solution were diluted with 900 μL of distilled water and centrifuged at 10,000 × *g* for 5 min. The supernatant was further clarified through ultrafiltration using an Ultrafree-MC with a molecular weight cutoff of 5000 Da (Merck Darmstadt, Germany) under centrifugation at 10,000 × *g* for 20 min. The filtrates were analyzed using ion chromatography (DX-300, Dionex, Sunnyvale, CA) equipped with an IonPac CS14 column and a cation self-regenerating suppressor (CSRS-I). The column was used for separation at room temperature. All were obtained from Dionex. The eluent was 10 mM methanesulfonic acid. The flow rate was 0.8 mL/min, and the detection was performed using electrical conductivity. A self-regenerating solution (water) was delivered to the suppressor under pressure at 15 psi.

### Preparation of diets

SPI was also suspended in water and freeze-dried in the same manner as HT-treated SPI without autoclaving and used as non-treated SPI. Experimental diets were prepared according to the recommendations by the National Institute of Nutrition in 1993 (AIN-93G). As shown in Table 1, experimental diets were prepared based on the AIN-93G formulation (20% protein). Casein in the standard AIN-93G formulation was replaced with either non-treated SPI or HT-treated SPI as the sole protein source in the experimental diets.

### Animal experiments

All animal experiments were conducted in accordance with institutional guidelines and were approved by the Animal Care Committee of Nara Medical University (approval nos. 12850, 12881, 13089, 13090).

Male C57BL/6J mice aged 3 weeks were purchased from Japan SLC (Hamamatsu, Japan). Four mice per cage were housed under standard laboratory conditions (23 °C, 55% humidity, and a 12-h light-dark cycle). Mice had access to a standard laboratory chow (CE-2, CLEA Japan, Tokyo) and water ad libitum for 1 week. After the acclimatization period, mice were randomly assigned to experimental groups and fed either the non-treated SPI diet (*n* = 12) or the HT-treated SPI diet (*n* = 12) for 6 weeks. Body weight was measured every 2 weeks. After 6 weeks of feeding experimental diets, behavioral tests were conducted. Two days after completion of all behavioral tests, all animals were fasted for 18 h with free access to water, and blood was collected from the inferior vena cava under anesthesia with isoflurane. Blood samples were allowed to clot and then centrifuged at 3000 × *g* for 10 min to obtain serum. Serum samples were stored at −80 °C until analysis.

For the single-administration experiment using HT-treated SPI and non-treated SPI, 7-week-old male C57BL/6J mice were purchased from Japan SLC. The mice were maintained on standard laboratory chow and water ad libitum for 5 weeks before the experiment. Thus, the mice were 12 weeks old at the time of administration. HT-treated and non-treated SPI suspensions in water (10%, w/v) were prepared by sonication. After overnight fasting for 18 h with free access to water, the mice were randomly assigned to experimental groups and administered HT-treated or non-treated SPI suspension at 1 g/kg body weight by oral gavage. Blood was collected from the inferior vena cava under isoflurane anesthesia at 30, 60, and 120 min after administration. All blood collections were performed as terminal sampling, with *n* = 4 mice per treatment group at each time point. Blood samples were allowed to clot and then centrifuged to obtain serum.

Baseline serum amino acid concentrations were determined using fasted mice before SPI administration. Because blood collection was performed under terminal sampling conditions, a single baseline group was used as a common pre-administration control rather than preparing separate baseline groups for each treatment condition. The baseline group consisted of four mice.

### Behavioral tests

Only one behavioral test was conducted per day. The next test was performed after at least 2 days. Each behavioral test was performed during the light phase, from 9:30 to 14:00. Mice were transferred to the test room at least 20 min before the test.

The open-field test was performed using an acrylic square box (40 cm in width, 40 cm in length, 30 cm in height). Mice were allowed to freely explore the field for 10 min. The center zone was defined as a 20 × 20 cm region in the center of the field. The number of entries into the center zone, time spent in the center zone, and locomotor activity in the whole field were measured using the TopScan LITE system (CleverSys, Reston, VA, USA).

The apparatus of the elevated plus maze test (O’Hara, Tokyo, Japan) consists of two open arms (25 cm in length, 5 cm in width, 0.5 cm in height) and two closed arms (25 × 5 × 16 cm), with a center platform (5 × 5 × 0.5 cm). The plus maze was elevated 50 cm above the floor. In this experiment, a mouse was placed in the center area of the maze with its head directed toward an open arm and allowed to explore the maze freely for 8 min. The number of entries into the open arm, time spent in the open arm, and the distance traveled were measured using the TopScan LITE system.

The object recognition test was performed using the same apparatus as the open-field test. The two sessions, acquisition and recognition, were conducted. A mouse was habituated to the field without objects for 1 h. During the acquisition session, two identical plastic balls (4 cm in diameter) were placed in the field, and the mouse was allowed to explore the objects for 10 min. After the acquisition session, the mouse was removed from the apparatus. The recognition session started 1 h after the acquisition session. Then, one of the objects was replaced with a column-shaped object (3 cm in diameter × 4 cm in height), which was considered the novel object, and the remaining ball object was considered the familiar object. The mouse was then placed inside the apparatus and allowed to explore the objects for 8 min. Close-range exploration was defined as behavior within 1 cm of the object. The time spent in close-range exploration of each object and the distance traveled in the field were measured using the TopScan LITE system. To assess recognition memory, an object recognition index was calculated by the following Eq. 1:

1 $${\rm{Object\; recognition\; index}}( \% )={{\rm{T}}}_{{\rm{Novel}}}/({{\rm{T}}}_{{\rm{Novel}}}+{{\rm{T}}}_{{\rm{Familiar}}})\times 100$$

where T_Novel_ and T_Familiar_ indicate the time spent in close-range exploration of the novel and familiar objects, respectively.

The three-chamber social test was performed according to protocols provided by the manufacturer (O’Hara). The apparatus consisted of three interconnected chambers: left, middle, and right chambers (each size, 20 cm in length × 40 cm in width × 35 cm in height) to allow free access. The left and right chambers had a wire cage to present a mouse. Following habituation to the apparatus without stranger mice for 5 min, the sociability test (session 1) and the social novelty recognition test (session 2) were sequentially conducted. In session 1, a stranger mouse (Stranger 1) was placed in the wire cage in the right chamber, and the subject mouse was allowed to freely explore all three chambers for 10 min. In session 2, Stranger 1 was moved to the wire cage in the left chamber, and a new stranger mouse (Stranger 2) was placed in the right chamber’s cage. The subject mouse was again allowed to explore the chambers freely for 10 min. Close-range exploration was defined as behavior within 1 cm of the wire cage. The chamber stay time and the close-range exploration time were measured using the TopScan LITE system. To evaluate social behavior, the following indices were calculated using Eqs. 2 and 3, respectively:

2 $${\rm{Sociability\; index}}( \% )={{\rm{T}}}_{{\rm{Stranger}}1}/({{\rm{T}}}_{{\rm{Stranger}}1}+{{\rm{T}}}_{{\rm{Empty}}})\times 100$$

3 $${\rm{Social\; novelty\; recognition\; index}}( \% )={{\rm{T}}}_{{\rm{Stranger}}2}/({{\rm{T}}}_{{\rm{Stranger}}2}+{{\rm{T}}}_{{\rm{Stranger}}1})\times 100$$

where T_Stranger1_, T_Stranger2_, and T_Empty_ represent the measured time for Stranger 1, Stranger 2, and the empty wire cage, respectively, using either chamber stay time or close-range exploration time depending on the analysis.

### Enzyme-linked immunosorbent assay

Serotonin (5-hydroxytryptamine) concentrations in the serum and brain from mice after the behavior test were measured in triplicate using a Serotonin ELISA kit (Abcam, Cambridge, UK) according to the manufacturer’s instructions. Brain tissues were collected without fixation, weighed, and homogenized on ice under acidic conditions, followed by centrifugation (15,000 × *g*, 15 min, 4 °C). The resulting supernatants were collected and diluted with the sample buffer prior to analysis.

### Statistical analysis

All data analyses were performed using GraphPad Prism Version 6.04 (GraphPad Software, San Diego, CA) or RStudio Version 4.4.1. Food chemical and in vitro digestion data are expressed as mean ± standard deviation (SD), based on three independent preparations (*n* = 3). Animal experiment data, including behavioral tests and serum biochemistry, are expressed as mean ± standard error of the mean (SEM), with biological replicate numbers indicated in the figure legends (typically *n* = 12 per group for behavioral assays). Differences between two groups (non-treated SPI vs. HT-treated SPI) were analyzed using the Student’s *t*-test when data were normally distributed, and the Mann–Whitney U test otherwise. For behavioral index data, chance level (50%) was evaluated using one-sample *t*-tests. Changes in contents of total amino acid and 3-methylhistidine in serum after administration of SPI or HT-treated SPI were compared using two-way ANOVA with treatment and time as factors. All statistical tests were two-sided, and a *P*-value < 0.05 was considered statistically significant.

## Supplementary information

Supplementary Information

## Data Availability

The source data underlying Figs. 1–9 and Supplementary Information are publicly available in Zenodo at https://doi.org/10.5281/zenodo.21301077.
