# Supplementary material for: High-temperature-treated soy protein isolate reduces amino acid availability and alters social novelty in mice
Source: NPJ Sci Food. 2026 Sep 3;10:324. doi: 10.1038/s41538-026-01091-0 (PMC13690705; doi:10.1038/s41538-026-01091-0)
Supplement: Supplementary file 1 [file 41538_2026_1091_MOESM1_ESM.docx]

**Supplementary Table S1. Composition of experimental diets in rats fed non-treated or HT-treated SPI.**

| Components (g) | Non-treated SPI group | HT-treated SPI group |
| --- | --- | --- |
| Casein | - | - |
| SPI | 13.5 | - |
| HT-treated SPI |  | 13.7 |
| Mineral mixture | 3.5 | 3.5 |
| Vitamin mixture | 1.0 | 1.0 |
| Corn oil | 5.0 | 5.0 |
| Cellulose | 4.0 | 4.0 |
| Alfa-corn starch | 48.7 | 48.5 |
| Sucrose | 24.3 | 24.3 |
| Total | 100 | 100 |

Diets were designed based on the nutrient balance of AIN-93G but modified to replace casein with either SPI or HT-treated SPI as the sole protein source.

**Supplementary Table S2. Body weight, food intake, and organ weights in rats after 2-week SPI or HT-treated SPI feeding.**

| Group | Non-treated SPI group | | | HT-treated SPI group | | |
| --- | --- | --- | --- | --- | --- | --- |
| Body weight (g) | 179.2 | ± | 3.75 | 170.3 | ± | 2.25 |
| Total body weight gain | 66.2 | ± | 2.43 | 55.5 | ± | 2.01^*^ |
| Total food intake (g) | 260.3 | ± | 8.22 | 246.8 | ± | 5.56 |
| Food efficiency ratio | 0.25 | ± | 0.00 | 0.23 | ± | 0.00^*^ |
| Liver weight  (g/ body weight of 100 g) | 3.69 | ± | 0.03 | 3.65 | ± | 0.01 |
| Pancreas weight (g/ body weight of 100 g) | 0.24 | ± | 0.01 | 0.22 | ± | 0.00 |
| Cecum weight  (g/ body weight of 100 g) | 1.39 | ± | 0.02 | 1.47 | ± | 0.01 |
|  |  |  |  |  |  |  |

Values are presented as mean ± SEM (n = 6).

Statistical significance was determined using the unpaired Student’s *t*-test (**P* < 0.05; compared with Non-treated SPI group).

Food efficiency ratio = body weight gain / total food intake.

| **Supplementary Table S3. Blood biochemistry analysis of rats after 2-week SPI or HT-treated SPI feeding.** | | | | | | |
| --- | --- | --- | --- | --- | --- | --- |
| Group | Non-treated SPI group | | | HT-treated SPI group | | |
| AST (IU/L) | 79.6 | ± | 5.02 | 92.3 | ± | 1.31 |
| ALT (IU/L) | 50.2 | ± | 1.55 | 51.5 | ± | 1.51 |
| Glucose (mg/dL) | 145.6 | ± | 1.59 | 146.7 | ± | 2.0 4 |
| Protein (mg/dL) | 5.58 | ± | 0.03 | 5.33 | ± | 0.02^*^ |
| Albumin (mg/dL) | 2.50 | ± | 0.02 | 2.42 | ± | 0.01 |
| Triglycerides (mg/dL) | 103.2 | ± | 4.33 | 80.5 | ± | 4.44 |
| Cholesterol (mg/dL) | 72.8 | ± | 0.98 | 73.0 | ± | 0.93 |
| Creatinine (mg/dL) | 0.20 | ± | 0.00 | 0.20 | ± | 0.00 |
| BUN (mg/dL) | 10.3 | ± | 0.08 | 11.5 | ± | 0.12^*^ |
| Uric acid (mg/dL) | 1.24 | ± | 0.02 | 1.05 | ± | 0.02 |

Data are presented as mean ± SEM (n = 6).

Statistical significance was determined using the unpaired Student’s *t*-test (**P* < 0.05; compared with Non-treated SPI group).

All measurements were outsourced to Oriental Yeast Co., Ltd. (Osaka, Japan).

|  | Contents in HCl hydrolysate (µmol/g of insoluble fraction) |
| --- | --- |
| Asp | 331.972 |
| Glu | 359.405 |
| Ser | 246.527 |
| Gly | 447.871 |
| His | 71.8139 |
| Arg | 164.02 |
| Thr | 222.706 |
| Ala | 543.958 |
| Pro | 204.571 |
| Tyr | 99.8223 |
| Val | 373.172 |
| Met | 67.6778 |
| Ile | 274.631 |
| Leu | 516.391 |
| Phe | 210.68 |
| Lys | 160.621 |

**Supplementary Figure S1. Amino acid composition of insoluble fraction after in vitro digestion of HT-treated SPI.**

Insoluble material obtained after pepsin-pancreatin digestion of HT-treated SPI was hydrolyzed with HCl and analyzed for amino acid composition.

Body Weight (g)

|  | Non-treated | HT-treated |
| --- | --- | --- |
| 1 w | 18.6708 | 18.3882 |
| 3 w | 23.3242 | 24.1736 |
| 5 w | 24.41 | 25.7455 |
| 7 w | 25.1567 | 26.3155 |

**Supplementary Figure S2. Body weight of mice during the six-week dietary intervention.**

Body weight was measured every two weeks. Data are presented as mean ± SEM (n = 12 per group). Statistical significance was determined using the unpaired Student’s *t*-test.
